# Sex determination systems in reptiles are related to ambient temperature but not to the level of climatic fluctuation

**DOI:** 10.1186/s12862-020-01671-y

**Published:** 2020-08-17

**Authors:** Paola Cornejo-Páramo, Andrés Lira-Noriega, Ciro Ramírez-Suástegui, Fausto R. Méndez-de-la-Cruz, Tamás Székely, Araxi O. Urrutia, Diego Cortez

**Affiliations:** 1 Center for Genomics Sciences, UNAM, CP62210, Cuernavaca, Mexico; 2 Milner Centre for Evolution, Department of Biology and Biochemistry, University of Bath, Claverton Down, Bath, BA2 7AY UK; 3 CONACYT Research Fellow, Red de Estudios Moleculares Avanzados, Instituto de Ecología, A.C. Carretera antigua a Coatepec 351, Col. El Haya, Xalapa, Veracruz Mexico; 4 Biology Institute, UNAM, CP04510 Mexico City, Mexico; 5 Department of Evolutionary Zoology and Human Biology, University of Debrecen, Debrecen, H-4032 Hungary; 6 Institute of Ecology, UNAM, 04510 Mexico City, Mexico

**Keywords:** Reptiles, Sex determination systems, Temperature-dependent sex determination, Genotypic sex determination, Climate fluctuation, Ambient temperature, Geographic ranges, Breeding seasons

## Abstract

**Background:**

Vertebrates exhibit diverse sex determination systems and reptiles stand out by having highly variable sex determinations that include temperature-dependent and genotypic sex determination (TSD and GSD, respectively). Theory predicts that populations living in either highly variable or cold climatic conditions should evolve genotypic sex determination to buffer the populations from extreme sex ratios, yet these fundamental predictions have not been tested across a wide range of taxa.

**Results:**

Here, we use phylogenetic analyses of 213 reptile species representing 38 families (TSD = 101 species, GSD = 112 species) and climatic data to compare breeding environments between reptiles with GSD versus TSD. We show that GSD and TSD are confronted with the same level of climatic fluctuation during breeding seasons. However, TSD reptiles are significantly associated with warmer climates. We found a strong selection on the breeding season length that minimises exposure to cold and fluctuating climate. Phylogenetic path analyses comparing competing evolutionary hypotheses support that transitions in sex determination systems influenced the ambient temperature at which the species reproduces and nests. In turn, this interaction affects other variables such as the duration of the breeding season and life-history traits.

**Conclusions:**

Taken together, our results challenge long-standing hypotheses about the association between sex determination and climate variability. We also show that ambient temperature is important during breeding seasons and it helps explain the effects of sex determination systems on the geographic distribution of extant reptile species.

## Background

Although signalling pathways that regulate the development of the gonads are broadly conserved among vertebrates [1]_,_ a great number of sex determination systems have evolved to determine which individual becomes a male or a female. Understanding sex determination systems has implications not only for the evolutionary biology of sexes but for ageing, senescence, and health sciences [2]. Recent studies have found that adult sex ratios correlate with the type of sex-determination system in amniotes [3]. Thus, males and females carrying specific sex determination systems have a greater chance to die before their sex counterparts. In humans, for example, shorter life expectancies of men have been associated with its XY system [4], resulting in an increased risk of developing diseases [5, 6].

Vertebrates exhibit two broad categories of sex determination systems: genotypic sex determination (GSD) where specific genetic elements direct the development of the gonads and environmental sex determination where external cues, such as temperature (temperature-dependent sex determination or TSD), specify the individual’s sex [7]. GSD and TSD systems probably represent the endpoints of a continuum [8, 9] because several species of reptiles exhibit intermediate states where the signaling cascade governed by sex-linked genes is overridden by thermal-induced sex reversal [9–12].

GSD systems are common in vertebrates [7, 13]. We know that the random segregation of sex chromosomes generally produces a 1:1 offspring sex ratio in GSD species and, therefore, mothers invest equally into producing males and females [14]. TSD systems are found in some fish and many non-avian reptiles, such as crocodiles, tuatara, turtles, and various lizards, including geckos, scincids, anguimorphs, and acrodonts [13]. Many factors have been proposed that drive the evolution of TSD systems, including sexual dimorphism, unequal survival rates, different sex maturation ages, inbreeding avoidance, mating competition, sex-specific variation in hatchling phenotypes, etc. [15–22]. However, a general explanation for the evolution of TSD has remained hotly debated.

A theoretical framework known as the Charnov-Bull model [23] is the most accepted hypothesis addressing the evolution of TSD. This hypothesis proposes that when incubation temperatures differentially affect the fitness of male and female offspring, selection should favour a link between sex determination and incubation temperatures [23]. TSD is also favoured in patchy environments where, for example, nests laid in slightly warmer areas (exposed sites) would produce different sex ratios compared to nests laid in slightly cooler areas (shaded sites) [17]. A major disadvantage of TSD systems, however, comes from their susceptibility to show elevated sex ratio variations in fluctuating environments. The Charnov-Bull model proposes that regular year to year environmental fluctuations could only cause mild fluctuations in sex ratios of TSD species. Nevertheless, if environmental fluctuations increase, biases in sex ratios also increase, and, in this scenario, GSD systems would be selected [23].

It has been difficult to find empirical evidence supporting the Charnov-Bull model given that most non-avian reptiles with TSD show long life spans and late sexual maturation. The available data derives from a few short-lived species. In the agamid *Amphibolurus muricatus* (family *Agamidae*) TSD enhances offspring fitness by promoting early hatchling of females [24], since warm incubation temperatures boost embryonic development. Thus, females can grow larger body sizes, have higher survival rates and reach sexual maturity at age one [22]. The lack of competition between females from different generations enables younger females to reproduce during their first mating season and, therefore, their reproductive success is enhanced when females hatch earlier [22]. Conversely, males are aggressive and territorial and mating competition is intense and young males are unlikely to reproduce until later years, so the time of hatching in males is not under selection [22]. Remarkably, analyses of lifetime reproductive success in *A. muricatus* indicated that the fitness of each sex is maximized by the incubation temperature that produces that sex [25].

Besides, the snow skink *Niveoscincus ocellatus* (family *Scincidae*) shows both sex chromosomes [26] and female-biased offspring under elevated temperatures [27], specifically in a population living at low altitudes [26], in a presumably more stable environment. The evolution of a temperature-dependent system capable of overriding the activity of the sex chromosomes [28] has also been driven by the early birth of females [26] because females grow larger body sizes that would also increase their reproductive success during their first mating season. In contrast, the *N. ocellatus* population living at high altitudes, where the environment fluctuations are probably higher, has sex chromosomes [26] and no effect of ambient temperatures. Thus, at high altitudes, in a presumably more fluctuating environment, selection has acted to balance the populations’ sex ratio at the expense of larger reproductive females.

Similarly to *N. ocellatus*, the Atlantic silverside fish (*Menidia menidia*) has two different types of populations [29, 30], one with a TSD system located in southern and warmer areas showing larger breeding and growing seasons. In contrast, a second population with a GSD system inhabits northern and cooler areas where shorter breeding and growing seasons have probably disrupted the link between offspring fitness and specific incubation temperatures. Moreover, the empirical association between specific climatic conditions and sex determination systems is also supported by data from viviparous reptiles. These reptile species inhabit extremely cold regions [31] and are strongly associated with GSD [32]. However, a study on the association of sex determination systems, environmental factors, breeding season lengths, and life-history traits has not been conducted across a wide range of taxa.

Non-avian reptiles offer an ideal taxon to test predictions of sex determination models as they are globally distributed, and exhibit species with either TSD or GSD systems. Here, we evaluated the relationship between sex determination systems and environmental factors using biogeographic data from 213 non-avian reptiles. Specifically, we investigated two long-standing hypotheses: 1) That higher variation in temperature during breeding seasons, warmer climates, extended longevities, larger breeding seasons, and oviparity are selective agents of TSD systems [7, 19, 22, 23, 25, 26, 29, 33]. And 2) that higher interannual variation in temperature during breeding seasons, colder climates, shorter lifespans, shorter breeding seasons, and viviparity select against TSD systems [32, 34–36]. We also examined precipitation data as an additional environmental variable. Finally, we tested the fit of several hypothetical scenarios between ambient environment, life-history traits, breeding season length, and sex determination transitions using phylogenetic path analyses to explore the likely cascade of changes that led to the observed relationships.

## Results

### Climate and climatic fluctuations

**Fig. 1:**
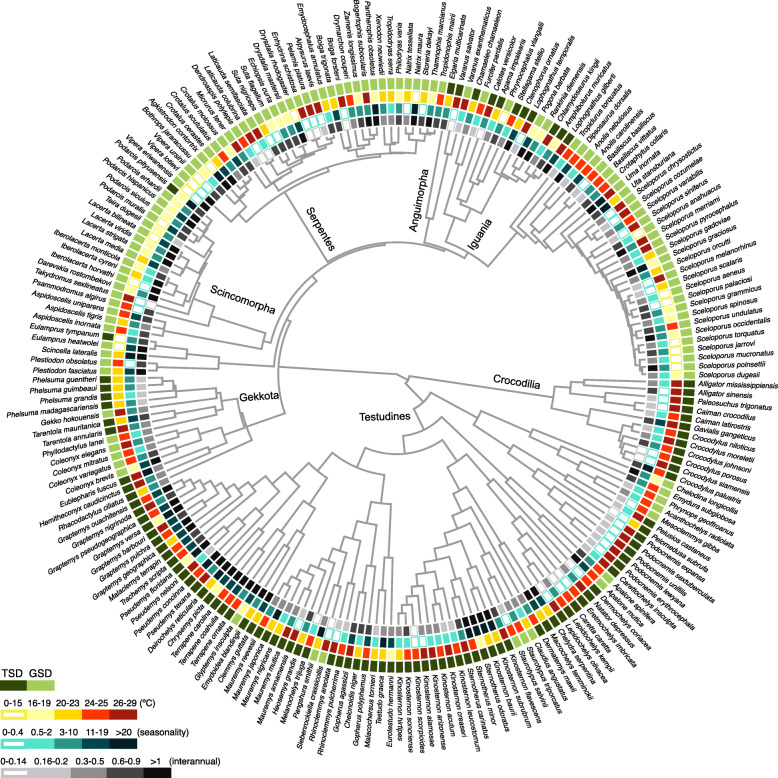
Phylogenetic relationship between sex determination systems and ambient temperature in reptiles. Relationships for 213 species with known breeding season (GSD = 112 species, TSD = 101 species). Light and dark green represent genotypic and temperature-dependent sex determination, respectively. Squares with colour gradient yellow-red indicate ambient temperatures over breeding seasons: very cold (0–15 °C), cold (16–19 °C), mild (20–23 °C), warm (24–25 °C), and very warm (26–29 °C), respectively. Squares with colour gradient turquoise-black indicate temperature variation during breeding seasons (seasonality). Squares with colour gradient grey-black indicate interannual temperature fluctuation over breeding seasons. Names in the phylogenetic tree correspond to reptile orders or infraorders

**Fig. 2:**
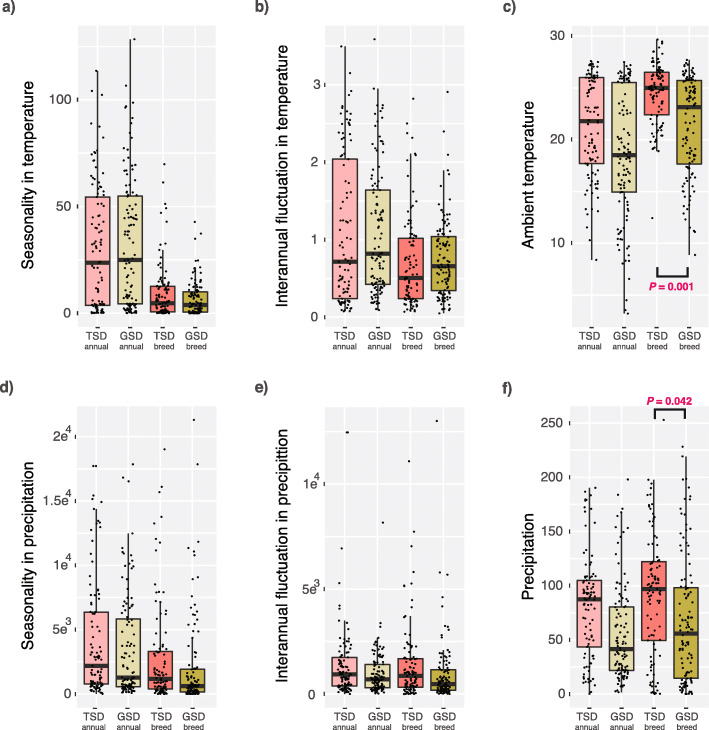
Climate and climatic fluctuations in reptiles with temperature-dependent and genotypic sex determination systems (*n* = 213 species). Boxplots representing (**a**) seasonality in temperature, and **b** interannual fluctuation in temperature, and **c** ambient temperatures for species with either TSD or GSD systems, based on annual (*annual*) and breeding seasons (*breed*) data. **d-f** Similar to a-c) but for precipitation data. Significant differences (Phylogenetic Generalized Least Squares test): exact *P* values are indicated. Error bars, maximum and minimum values, excluding outliers. See Additional file 2: Tables S1 and S2 for details. Temperature is given in Celsius. Precipitation refers to ml units of rain

We analysed 112 species with GSD and 101 species with TSD that represent 38 families and 11 independent transitions from GSD to TSD. We mapped 30 years of climatic data (temperature and precipitation) onto the geographical distributions of GSD and TSD species (Fig. 1; see Methods). We did not find differences between TSD and GSD in temperature variation during breeding seasons (i.e. seasonality) nor in interannual temperature fluctuation over breeding seasons (Phylogenetic Generalized Least Squares, PGLS, *P* > 0.05, *n* = 213 species; Fig. 2a, b).

Importantly, we found that sex determination systems are related to ambient temperatures because TSD species inhabit regions with significantly warmer temperatures during the breeding season (mean = 24.38 °C) than GSD species (mean = 21.39 °C; PGLS, *P* = 0.0011, slope = − 2.02, alpha = 15.7, *n* = 213 species; Fig. 2a). Moreover, average breeding season temperatures were less variable across species with TSD (interquartile range: 22.4–26.5 °C) than across species with GSD (interquartile range: 17.65–25.7 °C; ANOVA corrected by phylogeny -see Methods-, *P* < 0.001, *n* = 213 species; Fig. 2a). However, annual temperatures were not significantly different between the two classes of sex determination systems (PGLS, *P* > 0.05, n = 213 species). Overall, these results suggest that the association between climate and sex determination system is explained by TSD species breeding in periods with warmer temperatures.

Viviparous species are more likely to have GSD [32] and are also more likely to live in colder areas than oviparous ones [31]; a pattern we recapitulated in this study with 30 out of 31 viviparous species having GSD and a preference for colder climates (PGLS, *P* < 0.0001, slope = − 6.25, alpha = 14.9, *n* = 213 species; Additional file 1: Figure S1). To verify whether viviparity influenced the results, we constrained the analysis to oviparous species only. We did not find significant differences between GSD and TSD systems regarding temperature fluctuation (PGLS, *P* > 0.05, *n* = 182 species; Additional file 1: Figure S2). However, we recovered a significant association between TSD and warmer climates even when all viviparous species are removed from the analysis (PGLS, *P* = 0.018, slope = − 1.3, alpha = 11.4, *n* = 182 species -only oviparous species-, TSD = 97 species, GSD = 84 species; Additional file 1: Figure S2). Similarly, average breeding season temperatures were less variable across species with TSD (interquartile range: 22.81–26.5 °C) than across species with GSD (interquartile range: 19.52–25.81 °C; ANOVA corrected by phylogeny -see Methods-, *P* = 0.006, n = 182 species -only oviparous species-). Furthermore, we found that reproductive mode is not a significant confounding variable in explaining the association between sex determination system and the environment when both are included in the same model (PGLS, *P* = 0.0106 and *P* > 0.05, for SDS and SDS * reproductive mode, respectively; model used: ambient temperature ~ SDS + reproductive mode + SDS * reproductive mode, alpha = 16.9, *n* = 213 species).

**Fig. 3:**
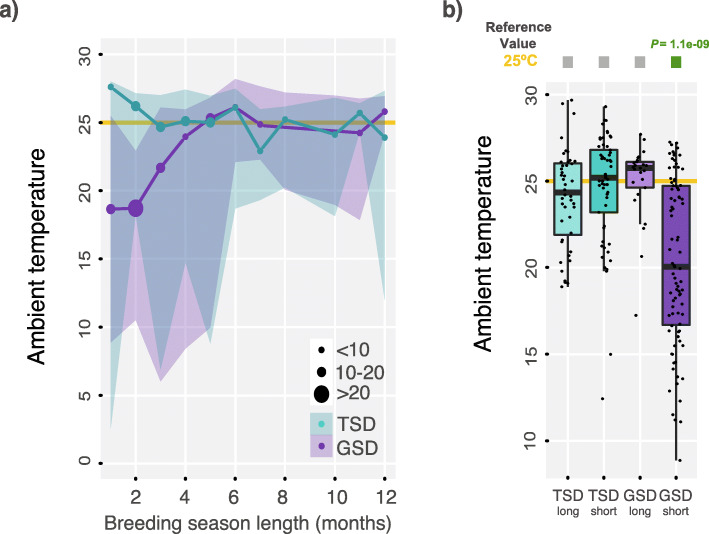
Ambient temperature in relation to the duration of the breeding season (n = 213 species). **a** Dots indicate the average ambient temperatures for species with breeding seasons of different lengths (measured in months). GSD species are shown in purple, whereas TSD species are shown in green. The number of species included in each category is indicated by the size of the dots. Shaded areas represent the annual temperature range (average maximum and minimum temperatures) for the species in each category. The yellow line at 25 °C indicates the approximate average ambient temperature for the majority of the groups. Temperature is given in Celsius. **b** Boxplots representing ambient temperatures associated with TSD or GSD species with either long breeding seasons (5–12 months) or short breeding seasons (1–4 months). Significant differences (Mann-Whitney *U* test): Benjamin Hochberg-corrected *P* < 0.05 of temperatures against a reference value of 25 °C (shared average ambient temperature for the majority of groups in panel a); grey filled squares denote non-significant differences, whereas green filled squares denote significant differences (significant *P* values are indicated). Error bars, maximum and minimum values, excluding outliers. Temperature is given in Celsius

We detected three different patterns associated with the length of the breeding season. First, we found that GSD species with short breeding seasons are located in areas where the temperatures are colder (ANCOVA corrected by phylogeny -see Methods-, *P* = 0.0032, n = 213; ANCOVA, *P* = 0.0015, *n* = 182 -only oviparous species-; model used: median temperature ~ SDS * breeding season duration; Fig. 3a and Additional file 1: Figure S2). Second, both GSD and TSD species with long breeding seasons (5–12 months) live in areas with warmer ambient temperatures (at around 25 °C; PGLS *P* > 0.05, *n* = 69 species; ANCOVA corrected by phylogeny, *P* > 0.05, n = 69 species, model used: median temperature ~ breeding season duration; Fig. 3a, b and Additional file 1: Figure S2). So to stay under optimal conditions, TSD species with short breeding seasons (1–4 months) reproduce close to the annual maximum temperatures also at approximately 25 °C, whereas GSD species with short breeding seasons are located in areas where the temperatures are colder (Fig. 3a, b and Additional file 1: Figure S2). Third, variations in temperature during breeding seasons are smaller than annual estimates (Fig. 2a), which could indicate a strong selection on the length of breeding seasons due to climatic factors. To further verify this hypothesis, we compared seasonality data against temperature variations from random subsets of consecutive months of the same length as the breeding seasons of each species. We found that estimates in temperature variation during breeding seasons for GSD and TSD species are significantly smaller than what you would expect by chance (Additional file 1: Figure S5).

We also examined precipitation data and we failed to find significant difference between GSD and TSD during breeding seasons (PGLS, *P* > 0.05, *n* = 213 species). However, TSD species breed in areas where precipitation is slightly higher (PGLS, *P* = 0.042, slope = − 19.1, alpha = 15.12, n = 213 species; Fig. 2d). Although this difference is lost when viviparous species are removed from the analysis (PGLS, *P* > 0.05, *n* = 182 species -only oviparous species-; Fig. 2d and Additional file 1: Figure S2) because viviparous species breed in areas with low precipitation, such as mountain summits. Consistently, we did not find differences in the range of precipitation between TSD and GSD species (ANOVA corrected by phylogeny, *P* > 0.05, n = 213 species; ANOVA corrected by phylogeny, *P* > 0.05, n = 182 species -only oviparous species-).

### Body size, longevity, and reproductive mode

**Fig. 4:**
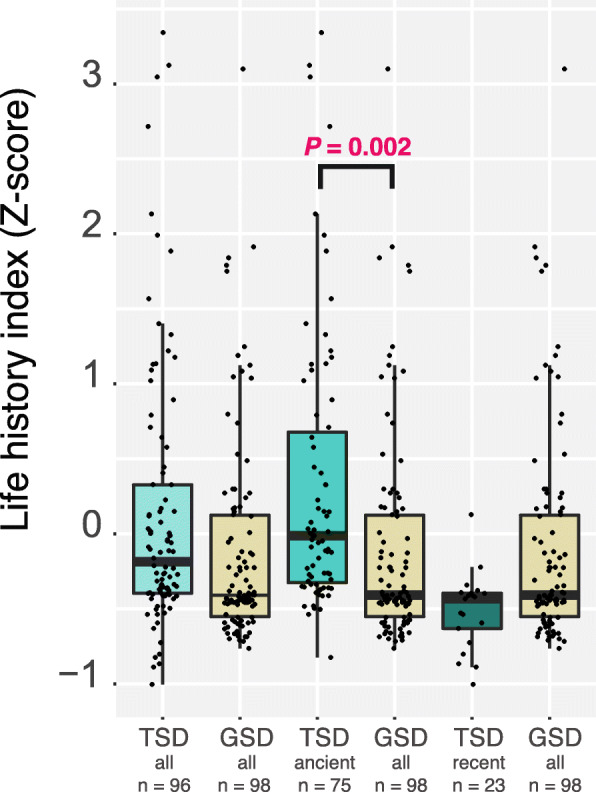
Life history traits in TSD and GSD reptiles. Boxplots representing the averaged life history index for all TSD or GSD species (*all*), for species that have conserved TSD systems for extended periods of time (turtles and crocodiles, −*ancient*-) and for species that have evolved more recent TSD systems (lizards, −*recent*-). The figures show Z scores. *P* values refer to Phylogenetic Generalized Least Squares test. Error bars, maximum and minimum values, excluding outliers. N refers to the number of species

We used two proxies that represent life-history traits on the fast-slow continuum: body size and longevity. Since only 46 species (21% of the species) have data for the three life-history traits (body length, body mass, and longevity) we transformed the life-history traits into Z scores and calculated an average Z index across the 3 traits to gain further statistical power. No significant differences were observed in life-history traits when comparing TSD and GSD species (PGLS, *P* > 0.05, *n* = 190 species). However, previous studies [33, 34] compared life-history traits in ancient TSD systems (i.e. TSD systems that derive from the last common reptilian ancestor) and recent TSD system (i.e. TSD systems that transitioned from GSD in specific groups), and found significant differences only for ancient TSD systems. We, therefore, sought to confirm this pattern using the combined life-history index. We examined crocodilians and turtles, representing ancient TSD systems, and found that these species were further toward the slow end of the continuum compared to GSD species (PGLS, *P* = 0.0104, slope = − 0.66, alpha = 2.24, *n* = 172 -all species-; *P* = 0.033, slope = − 0.57, alpha = 1.25, *n* = 146 -only oviparous species-; turtles with TSD or GSD were included in the analyses; Fig. 4). This association is likely driven by the extended longevities shown by turtles and crocodiles compared to squamates (PGLS, *P* = 0.0014, slope = − 0.92, alpha = 4.52, *n* = 91 -all species-; *P* = 0.0021, slope = − 0.91, alpha = 5, *n* = 84 -only oviparous species-; squamates with GSD and turtles with TSD or GSD were included in the analyses). Interestingly, lizards showing more recent TSD systems were not different from GSD species when the averaged life history index or the Z score for longevity were analysed (PGLS, *P* > 0.05, *n* = 119 -all species-; *P* > 0.05, n = 91 -only oviparous species-; Fig. 4).

### Phylogenetic path analyses

**Fig. 5:**
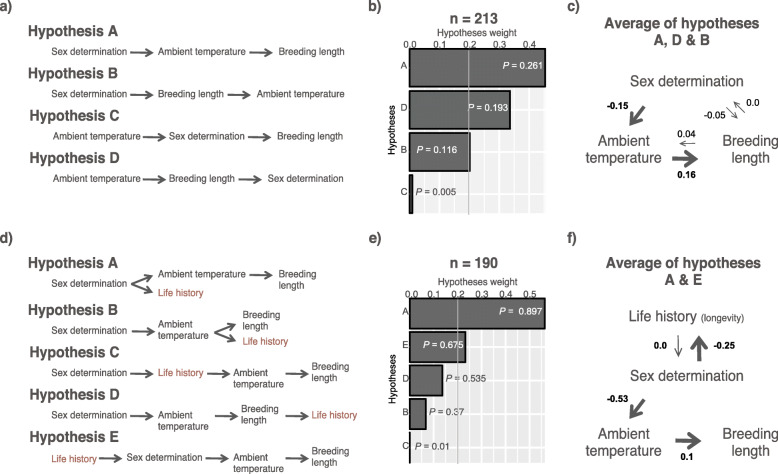
Phylogenetic path analyses of competing evolutionary theories of sex determination. **a** The four hypothesised scenarios (see Methods) were fitted using phylogenetic path analyses. **b** Histograms indicate the weight of each hypothesis, significance threshold, within 2 CICc, is indicated with a line. Bar labels are *P* values, significance indicates rejection of the model. *N* = 213 (full dataset). **c** Diagram summarizing the weighted graph obtained after the average of hypotheses A, D and B. Arrows indicate the hierarchical relationship across variables; the primary association is at the top of the diagram, whereas succeeding dependent variables are indicated by the direction of the arrows. Values of the relationships resulting from the phylogenetic path analyses are indicated next to the arrows. Significance of the association is indicated by the width of the arrows. **d-f** Similar to a-c) but for hypotheses including the averaged life history index. *N* = 190

To infer the most likely evolutionary transitions between the environment, the duration of the breeding season, life-history traits, and sex determination systems, we carried out phylogenetic path analyses [37]. We first tested the relationship between sex determination systems, ambient temperature, and the duration of the breeding season (the three main factors for which we found significant relationships) using the full dataset of species (*n* = 213 species; Fig. 5). We aimed to examine the directionality between ambient temperature and sex determination systems, and understand whether breeding season lengths were primarily influenced by sex determination or by ambient temperatures. We found that three models fit the data models A, D, and B (Fig. 5b). To obtain the final model we averaged models A, D, and B based on their specific weights (see Methods). The resulting final model supports that the type of sex determination system influences the ambient temperature at which the species reproduces and nests. This association then defines the duration of the breeding season (Fig. 5c).

Next, we sought to explore the relationship between life-history traits. We could not find a significant model when the three life-history traits were included in the analysis as independent variables, probably due to limited data (*n* = 46 species; 14 GSD and 32 TSD). We, therefore, used the averaged life history index to test five different hypotheses (*n* = 190 species; Fig. 5d). Models A and E fit the data (Fig. 5b). The final (averaged) model indicated that the sex determination system also influences life-history traits. We found a similar pattern when the Z score for longevity was used instead of the averaged life history index (*n* = 103 species).

Overall, the path analysis reinforced the results shown in Figs. 1, 2, 3 and 4 and provided the directionality in the interactions, supporting an important connection between sex determination systems, the environment, the length of the breeding seasons, and other life-history traits.

## Discussion

In this study, we collected 30 years of environmental data (temperature and precipitation) and mapped these variables to the geographical ranges of non-avian reptiles with a known breeding season, representing 11 independent transitions from GSD to TSD (Fig. 1). Importantly, we failed to find broad support for the two hypotheses we tested. Specifically, we found that a key predictor proposed for sex determination systems, that is, the amount of climatic fluctuation within a species’ geographical range [19, 22, 23, 25], was not significantly different between TSD and GSD species. In other words, we found similar levels of temperature variation during breeding seasons and in interannual estimates over breeding seasons. These results imply that TSD and GSD species are confronted with similar temperature fluctuations and that GSD systems are not inevitably selected in more unstable environments. This could also explain why non-avian reptiles with TSD show worldwide distributions (Additional file 1: Figure S3).

Our results supported four specific relationships. First, we found a strong association between sex determination systems and ambient temperatures. Specifically, we found that GSD systems have allowed non-avian reptile species to invade a wider range of ecoregions showing both warm and cold temperatures, whereas species with TSD systems reproduce preferentially in warmer environmental conditions. This is a general adaptative trait associated with the evolution of TSD that is consistent in both long-lived species with TSD (e.g. turtles and crocodiles; species with great overlap across generations) and short-lived species with TSD (e.g. geckos and acrodonts; species with limited overlap across generations). Temperatures are generally more stable over the years (compared, for example, to precipitation) and, therefore, non-avian reptiles could adapt to them to sense optimal environmental cues for their survival. Second, our data indicate that a major adaptation to colder climates has been the evolution of shorter breeding seasons, as previously observed in the Atlantic silverside fish [29, 30]. TSD, for example, mates and nests in places where the ambient temperature is always close to 25 °C regardless of the duration of the breeding season, indicating that ~ 25 °C probably represents optimal conditions for nesting, sex-determination, and embryo development. We propose that selection has acted so that non-avian reptile species reproduce preferentially during the warmer and less variable months of the year with strong selection acting on the length of breeding seasons depending on the biogeographical distribution of the species. Third, our data confirm that viviparous species are strongly associated with GSD [32] and their distributions are limited to cold areas [31]. Four, we found a correlation with precipitation that is conditioned by reproductive mode.

Among turtles and crocodilians, TSD is inferred to originate early in the reptile phylogeny [33]. TSD to GSD transitions in Squamata occurred over a large evolutionary period spanning over 300 million years with very early transitions as in *Pleurodonta* and *Anguimorpha*, to more recent ones as in *Gekkota* and *Acrodonta*. Remarkably, a recent study found similarities in the temperature-dependent molecular mechanisms of sex determination in a turtle (*Trachemys scripta*), a crocodile (*Alligator mississippiensis*), and an acrodont (*Pogona vitticeps*) [38]. One could argue, therefore, that TSD was maintained in non-avian reptiles under low levels of selection and with no current adaptative significance [18]. Moreover, recent findings suggested that the evolution of TSD was not adaptative because, in reptiles that exhibit GSD with thermal-induced override, the sex chromosomes could be lost under climatic shifts, resulting in a GSD to TSD transition with no adaptative cost [10, 12, 39, 40]. Our findings are at odds with these non-adaptative hypotheses because we found a significant association between sex determination systems and ambient temperature.

Furthermore, numerous adaptative hypotheses have been proposed to explain the evolution of TSD systems; many of which suggest that the differential fitness of male and female offspring is linked to a higher variation in temperatures during breeding seasons and that GSD systems should be selected in more unstable environments [7, 19, 22]. As mentioned above, our results failed to support these relationships. We propose that temperature fluctuations across interannual breeding seasons may result in shifting proportions of male and female offspring, which, overall, could balance the adult sex ratio in species showing some overlapping generations [34–36].

Interestingly, a more recent hypothesis, known as the ‘survival-to-maturity’ hypothesis [41], proposed that the combination of sex-specific survival and sex-differential age at maturity can drive the evolution and maintenance of TSD. This hypothesis has received support from a recent comparative study [15]. We note that our results are not in conflict with the predictions made by the survival-to-maturity hypothesis, which, indirectly, seem to support the model.

Furthermore, the phylogenetic path analyses show that sex determination systems influence the ambient temperature at which the species reproduces and nests. This interaction is the primary association that would then influence other variables, such as the duration of the breeding season and life-history traits. The results from the phylogenetic path analyses are at odds with an adaptative hypothesis where environmental changes select for specific traits in the species. We think a possible explanation for this result could be linked to the significant association between TSD species and warmer temperatures. Recent work found that TSD species could be more successful in female-favouring temperatures than in male-favouring temperatures [42–47]; female-favouring temperatures are warm temperatures for most species. Thus, the type of sex determination system could help maintaining optimal conditions for the species by influencing the geographic range of the species and their breeding season length.

GSD systems are predominant in fish, amphibians, reptiles, birds, and mammals, suggesting that genotypic-based sex determination systems offer an advantage over the environmental ones. Indeed, transitions from environmental sex determination to genotypic sex determination have been more frequent in fish and non-avian reptiles alike [48], probably due to the emergence of genetic elements that restored balanced sex ratios in TSD species with extreme sex ratios. The association with life-history traits is likely driven by turtles and crocodiles, in agreement with previous reports [33, 34], and provides little insights into the evolution of sex determination systems. These lineages have evolved prolonged longevities [33] and other life-history traits (i.e. delayed reproductive maturity). It is, therefore, not surprising that they have also maintained TSD systems for extended periods of time.

## Conclusions

The present study represents the most ambitious assessment to date of the link between ambient temperature and sex determination systems. The data set represents a systematic effort to compile data for all non-avian reptile species with available breeding season, climate, and sex determination system. Although we controlled for various potential sampling biases, including the uneven sampling across continents, we recognise that there are limited data from some regions of the world such as Central and Southern Africa or China since reptiles in these regions appear to be understudied. This could result in an under-representation of squamate species inhabiting particular ecoregions, for example, tropical environments. However, association-based phylogenetic analyses such as PGLS are robust to uneven sampling [49]. Nevertheless, additional data collected in the future on sex determination systems and breeding season length will allow extending the scope of our analyses by focusing on specific ecoregions and/or investigating specific groups such as *Squamata* or *Testudines*.

Overall, our results shed light on the relationships between sex determination systems, climate, breeding seasons, and life history-traits using the largest dataset of non-avian reptiles available to date. Our results warn of a potentially alarming scenario under the current climate change for species that have short breeding seasons and may struggle to adapt to environmental stresses. It would be important that future conservation studies of reptile species, such as ref. [50], pay special attention to this particular type of reptiles with TSD that could be at a higher risk.

## Methods

### Data collection

We collected the full list of reptiles with known sex determination system from the Tree of Sex database [13]. We divided the sex determination systems of the species in two categories: genotypic sex determination (GSD) and temperature-dependent sex determination (TSD). We then searched the literature and dedicated reptile databases for the duration and month intervals of the breeding seasons for all reptile species with known sex determination systems. The detailed records for the species with collected information and their corresponding references are listed in Additional file 2: Tables S1 and S2. When multiple breeding seasons where found for a given species, the consensus period was selected. Several species names reported in the Tree of Sex database have changed (the species have been reclassified) and did not follow the current species classification (e.g. different genus). Therefore, we worked with all synonyms obtained from the RedList database (http://www.iucnredlist.org/; version 3), the Reptile Database (http://www.reptile-database.org/) and the Mexican Collection of Reptiles (http://www.ib.unam.mx/zoologia/#colecciones-zoologicas-nacionales) to solve the inconsistencies. We only used the current names for each species in strict agreement with the names used in the RedList database. The list of synonyms can be found in Additional file 2: Tables S1and S2. Lastly, we downloaded the shapefiles of their distributions from the RedList database (http://www.iucnredlist.org/; version 3). For 128 species we successfully collected the sex determination system, the shapefile of their geographical distribution and the breeding seasons in months (Additional file 2: Table S1). The mass and size values represent averages for both sexes. Two species, *Sceloporus aeneus* and *Lacerta vivipara*, have reports of oviparous and viviparous populations, we chose the main population’s reproduction mode (viviparity). Temperature and precipitation data from the entire surface of the planet were downloaded from the Climatic Research Unit (http:// http://catalogue.ceda.ac.uk/uuid/3df7562727314bab963282e6a0284f24; version 3.24.01), a database that has month-by-month variations in climate over the period 1901–2015, on high-resolution (0.5 × 0.5 degree) grids.

### Generation of distributional ranges for additional species

Out of the 128 species mentioned above, only the 22% show TSD. We decided to generate additional shapefiles for species for which we also collected data on their breeding season. We generated shapefiles for 85 additional species, thus bringing the total number of analysed species to 213 (128 species with RedList shapefiles plus 85 extra species). Of these, we generated shapefiles for 72 species with TSD. Given that, most of these additional species were turtles (64 species of turtles; 8 species from *Squamata*); we also generated shapefiles for 13 turtle species with GSD. In order to characterize the species’ geographic ranges, we implemented ecological niche modeling routines using the maximum entropy algorithm in Maxent [51]. This was done by searching for best candidate models using the R package kuenm developed by Dr. Marlon E. Cobos (https://github.com/marlonecobos/kuenm). We first compiled the species’ occurrences through several databases including the Global Biodiversity Information Facility (GBIF; https://www.gbif.org/), Biodiversity Information Serving Our Nation (BISON; https://bison.usgs.gov/#home), Berkeley Ecoinformatics Engine (Ecoengine; https://ecoengine.berkeley.edu/), Integrated Digitized Biocollections (iDigBio; https://www.idigbio.org/tags/database), Atlas of Living Australia (ALA; https://www.ala.org.au/), iNaturalist (https://www.inaturalist.org/), VertNet (http://vertnet.org/), and Ocean Biogeographic Information System (OBIS; http://www.iobis.org/). All the occurrences per species were cleaned from obvious georeferencing errors or taxonomic misidentifications through careful inspection in a GIS (Geographic Information System) and based on information gathered from The Reptile Database, which contains updated taxonomic information (http://reptile-database.reptarium.cz/). The number of occurrences per species ranged between 1 and 13,192, although the majority (76 species; 89.4%) had more than 25 occurrences. Only nine species had less than 12 occurrences and were treated as data deficient species; for five of these data deficient species we decided to use alternative geographic range estimations rather than the outcome of a niche model (see below) as follows: for three of these data deficient species (*Mauremys nigricans*, *Podocnemis erythrocephala*, *Siebenrockiella crassicollis*) we decided to use the ecoregions [52] that intersected with their occurrences as a proxy of their distribution after carefully inspecting that these ranges corresponded to the species known distributions, and for two other data deficient species (*Phelsuma guentheri*, *Phelsuma guimbeaui*) that are located in the Mauritius island, we used the polygon of the island as a proxy of their geographic range. For the rest of the data deficient species (*Geochelone elephantopus*, *Mauremys annamensis*, *Pangshura smithii*, *Eurotestudo hermanni*) we conducted niche modelling as described as follows. For the species with more than 12 occurrences, the points representing the locations of each species were then used to intersect the terrestrial ecoregions of the world [52], to which we then added a 1-degree buffer to use as the accessibility area [53]; this area was implemented in order to mask the environmental layers that were then needed to calibrate the models. All occurrences were spatially filtered with a 20 km radius to avoid spatial autocorrelation and overfitting the models. Models were calibrated with the seven least correlated bioclimatic layers from the WorldClim database (http://www.worldclim.org/) at a spatial resolution of 2.5 min: Annual Mean Temperature; Mean Diurnal Range (Mean of monthly; max temp - min temp); Max Temperature of Warmest Month; Min Temperature of Coldest Month; Annual Precipitation; Precipitation of Wettest Month; Precipitation of Driest Month. Candidate models were explored setting up the maximum entropy algorithm in Maxent [51] in the R package kuenm [54] (https://github.com/marlonecobos/kuenm) with all possible combination of features and regularization multipliers of 0.1, 0.5, 1, 2, 3, and 4. This generated 348 models per species, which were then subjected to evaluation to select the best model parameterization based on statistical significance according to the partial Receiver Operative Characteristic [55] (ROC), omission rate [55] (i.e., a user-selected proportion of occurrence data that may present meaningful errors), and model complexity (Akaike Information Criterion for small samples (AICc)). Evaluation parameters were set to 10% for omission rate, with 50% randomly sampled occurrence points from the test data for bootstrapping, and 500 iterations for bootstrapping; such procedure produced a table with the best models corresponding to those with highest mean AUC ratio from the partial ROC, lower omission rate and AICc, as well as number of parameters per model. When more than one best model was obtained, we selected the best one based on the highest value of the AUC mean ratio. Finally, the specific parameterization from the best model was used to re-run the Maxent procedure and generate the final model, which represented the best estimate of the species’ geographic range. The minimum criteria regarding the outcome of model evaluation was the statistical significance of the model (*P* < 0.001) and an AUC ratio > 1.4, considering that values of AUC ratio that depart upwards from one perform better than random [55]. The final model per species was based on the raster of the median of 10 model projections of that best model in each species accessible area. To depict the geographic potential distribution of each species, each raster was thresholded based on the 10-percentile training presence. The geographic projection of the resulting binary map was then converted to a shapefile. The resulting shapefiles for 85 reptile species are available in the figshare platform at the following link https://figshare.com/s/83d59b0d26f8df636621. For these additional 85 species, we also collected information regarding their reproductive mode and life history traits. The detailed records for these species are listed in Additional file 2: Table S2.

### Mapping climate data to the species distribution

Shapefiles downloaded from the RedList database (http://www.iucnredlist.org/; version 3) and shapefiles generated for this project contain polygons with geographical coordinates (latitude and longitude) representing the species distribution. The environmental data from CRU (http:// http://catalogue.ceda.ac.uk/uuid/3df7562727314bab963282e6a0284f24; version 3.24.01) has climate surfaces gridded at a spatial resolution of 0.5 × 0.5 degrees. We matched the climate data with the species shapefiles using a dedicated R package built by Dr. Anna Krystalli as part of the Newton Advanced Fellowship program. The R package used in the study is available at https://github.com/annakrystalli/IUCNextractR. Briefly, the R package extracted the climate grids that overlap with the species’ polygons and returned, for each of the 213 shapefiles, the average monthly temperature for a given time period. In our case we selected a 30-year time period of climate data, 1960–1990. To verify the correctness of the method, we manually mapped onto the surface of the world the shapefiles and average temperatures for the 213 reptile species with known breeding season (Additional file 1: Figure S3; the species’ shapefiles were layered onto the world map obtained from the “rworldmap” R package [56]). Species with TSD and GSD are globally distributed on all continents, and the observed patterns are supported by combination of data from various continents (Additional file 2: Table S3). For the 213 species, the beginning and end months of the breeding season were recorded in a numerical format, where 1 was January, 2 was February, etc., until 12 represented December. For the one species with not known end of the breeding season, we defined the end of the breeding season as December. We then recovered the median temperature (ambient temperature) and precipitation (precipitation) of all months comprised in the breeding season for 1960–1990. We also used the data to calculate the seasonality in temperature (i.e. average of the variances of the breeding season months of each year) and the interannual fluctuation in temperature (i.e. average of the variances of each month of the breeding season through the years). For the six marine turtles and the six marine snakes, shapefiles are limited to the coasts where they nest or the coastlines where they live, respectively.

### Phylogenetic generalized least squares analyses

The analyses were performed using a final dataset of 213 species with known breeding season (128 species with shapefiles from RedList plus 85 species with shapefiles we generated). This dataset of 213 species was composed of 112 species with GSD and 101 species with TSD, representing 38 families and 11 independent transitions from GSD to TSD. The transitions were estimated based on previous results reported in ref. [33, 57].

Sex determination systems and reproductive modes data were converted to binary format, where TSD was 1, GSD was 0, oviparous species were 0 and viviparous species were 1. We modelled the sex determination systems and the reproductive mode as a function of the ambient temperature, precipitation, seasonality in temperature and precipitation and between years fluctuation in temperature and precipitation over annual estimations and breeding season by means of Phylogenetic Generalized Least Squares (PGLS) approach [58]. We used the “gls” (Fit Linear Model Using Generalized Least Squares) function in the “nlme” R package, which implements GLS models accounting for phylogeny through maximum likelihood estimations considering that the response variables evolved following an Ornstein-Uhlenbeck process, that is, the traits we measured are the result of natural selection rather than random processes occurring along the species phylogeny. Similar results were obtained using the phylogenetic logistic regression [59] implemented for binary dependent variables in the “phyloglm” function in the “phylolm” R package, method = “logistic_IG10”, btol = 10–50. Results obtained with the phylogenetic logistic regression can be found in Additional file 2: Table S3. Reptile species show a large range of geographical distributions and, consequently, species with small geographical ranges may have more precise environmental estimates than species with large distributions. Thus, climatic estimates averaged across a species’ distribution may not be representative of the climatic conditions experienced by the entire population [60]. To control for geographic range, we supplied the “weights” parameter in the PGLS function. To do so, we first log_10_ transformed the species’ area sizes. We then defined the specific weights for each species such as: 1/(*smallest_area_size_in_the_dataset*/*the_species_area_size*). This results in an inverse proportion where the species with the largest areas have the lowest weights in the PGLS. Variance in ambient temperature within the species’ distributions increases as the geographic range increases (Additional file 1: Figure S4). Limiting the analysis to breeding seasons significantly reduces the uncertainties in the estimates associated with the geographic range of the species (Additional file 1: Figure S4). We found that the association between sex determination systems and ambient temperatures was still significant after correcting for the size of the geographical ranges (weighted-PGLS, *P* = 0.0114, slope = − 1.37, alpha = 12.8, *n* = 182 species -only oviparous species-). The species phylogenetic tree used in the analyses derived from the combination of the curated phylogeny for the order *Squamata* obtained from ref. [61] and the phylogeny of *Testudines* (turtles), *Rhynchocephalia* (tuatara) and *Crocodilia* (crocodiles) obtained from ref. [62] and complemented using the Timetree database (http://www.timetree.org/). Branch lengths were adjusted to match the scale used in the tree from ref. [61]. We tested for the phylogenetic signal contained in our variables based on the species’ tree and using the “phylosig” function, “lambda” method, in the “phytools” R package [63]. Sex determination systems, phylogenetic signal 1.003, *P* = 1.65e-69; Reproductive mode, phylogenetic signal 0.72, *P* = 3.11e-17; Ambient temperature, phylogenetic signal 0.437, *P* = 3.83e-09; Temperature seasonality, phylogenetic signal 0.35, *P* = 0.001; Interannual temperature variation, phylogenetic signal 0.51, *P* = 6.31e-06; Precipitation, phylogenetic signal 0.07, *P* = 0.011; Precipitation seasonality, phylogenetic signal 1.003, *P* = 1.8e-15; Interannual precipitation variation, phylogenetic signal 0.82, *P* = 1.07e-07.

Generally, biases in the datasets are measured, and accounted for, by analysing hundreds of alternative tree topologies gathered in large phylogenetic projects in other lineages, such as the Bird Tree Project [64]. However, these approaches cannot be performed in the reptilian clade given the lack of alternative phylogenies. The species tree in ref. [61] is the most complete tree available for reptiles. We thus tested for potential biases in the dataset using two alternative approaches. First, we tested for potential biases introduced by the uneven sampling of reptiles across continents. We carried out PGLS analyses where one continent at the time was not considered (Additional file 2: Table S3). Secondly, we tested for potential biases introduced by species with unique attributes by repeating the PGLS analyses for all variables using a decreasingly random number of species, starting at 90% of the 213 species with known breeding season, and ceasing the analyses when we reached 60% of the 213 species. We completed 10 replicates for each category (Additional file 2: Table S3). The only restriction in the analyses was that each of the 38 reptile families was represented by at least one species.

### Additional statistical analyses and graphics

Additional statistical tests were performed using the R package, standard libraries. Data was plotted using the R package, “ggplot2” library [65]. Figure 1 and Additional file 1: Figure S1 were plotted using the “ape” and “phytools” R packages [63]. To test if there were differences in temperature and precipitation ranges, we used the ANOVA test corrected by phylogeny within a PGLS, using the “anova” test from the “stats” R package contrasting two generalized least squares (gls) models we obtained using the “gls” function from the “nlme” R package. That is, we contrasted a null model against an alternative model where the variation on temperature and precipitation that is not explained by the phylogeny is free to fluctuate across groups of species depending on the type of SDS or reproductive mode. For the null and alternative models, we first calculated a generalized least squares (gls) model with the maximum likelihood method (ML). Then, the two models were contrasted with the ANOVA test. Lastly, in order to test the association between the environmental variables (temperature and precipitation), the duration of the breeding season, and the SDS or reproductive mode, we use an ANCOVA tests implemented with PGLS. The model used was: resulting_model <− median ambient variable ~ sex determination system + breeding season duration + sex determination system * breeding season duration.

### Phylogenetic path analyses

Phylogenetic path analyses [37] were performed using the “phylopath” R package [66], model = “OUfixedRoot”, method = “logistic_MPLE”. We first defined four hypotheses to be tested with the full data set of 213 species. Hypothesis A assumed that the sex determination systems influenced ambient temperature, which in turn influenced breeding season length. Hypothesis B assumed that sex determination systems influenced breeding season length and then ambient temperature. Hypothesis C assumed that the ambient temperature influenced sex determination systems, which in turn influenced breeding season length. Finally, hypothesis D assumed that ambient temperature influenced first the breeding season length followed by sex determination systems. We did not assess breeding season length as an independent variable. Similarly, given that GSD was shown to precede viviparity [32] and is confounded with cold climates, we did not test models where reproductive mode was a major evolutionary constraint. In a second set of hypotheses we included life history traits, first as independent variables, but also as a transformed Z score. Since body mass, body length and longevity correlate with each other (Additional file 1: Figure S6), prior to run the phylogenetic path analyses, these three variables were transformed into a single Z score (scores are listed in Additional file 2: Tables S1 and S2). Z scores were obtained based on the following formula: Z_i_ = (x_i_ – x̄)/*s*; where x_i_ represents the values of the variable, x̄ represents the mean of the variable and *s* represents the standard deviation of the variable. For each species, a unique life history index was obtained by calculating the average of the body length Z score, body mass Z score and longevity Z score. When more than one model was supported by the data, we produced an average of the models based on their specific weights using the *average* function and by specifying the *avg_method = “full”* parameter. We also ran the analyses including the reproductive mode, however, given the strong association between viviparity, GSD, and cold climates (almost 100% of the viviparous species are GSD and live in cold areas), the path analyses made these associations the main drivers of the analyses, thus clouding all other associations.

## Supplementary information

**Additional file 1: Figure S1.** Phylogenetic relationship between reproductive mode and ambient temperature (N = 213 species). **Figure S2.** Climate and climatic fluctuations in reptiles with temperature-dependent and genotypic sex determination systems for oviparous species only (n = 182 species), and ambient temperature in relation to the duration of the breeding season for oviparous species only (n = 182 species). **Figure S3.** World maps showing the distributions and average temperature of species with GSD or TSD and known breeding seasons. **Figure S4.** Ambient temperature and the variance within the geographic range of the species relative to the size of the geographic range of the species. **Figure S5.** Seasonality data from reptiles (n = 213) compared to random data. Figure S6. Correlations between continuous life history traits.

**Additional file 2: Table S1.** Data for 128 reptile species with known breeding season and Redlist shapefiles used in this study. **Table S2.** Data for 85 species with known breeding season and projected shapefiles. **Table S3.** Controls for potential biases and results of the phylogenetic logistic regressions.

## Data Availability

All data used in this study are found in the Supplementary Tables. The R package used to combine the climate data and the species shapefiles is available at https://github.com/annakrystalli/IUCNextractR. The R package used for the ecological niche modeling routines is available at https://github.com/marlonecobos/kuenm. Generated shapefiles for 85 reptile species are available in the figshare platform at https://figshare.com/articles/Reptile_shapefiles/7416638.

## Abbreviations

ANCOVA: Analysis of Covariance
ANOVA: Analysis of Variance
GSD: Genotypic Sex Determination
PGLS: Phylogenetic Generalized Least Squares
TSD: Temperature-dependent Sex Determination
