# Supplementary material for: Sex determination systems in reptiles are related to ambient temperature but not to the level of climatic fluctuation
Source: BMC Evol Biol. 2020 Aug 17;20:103. doi: 10.1186/s12862-020-01671-y (PMC7433102; doi:10.1186/s12862-020-01671-y)
Supplement: Supplementary file 1 — Additional file 1: Figure S1. Phylogenetic relationship between reproductive mode and ambient temperature (N = 213 species). Figure S2. Climate and climatic fluctuations in reptiles with temperature-dependent and genotypic sex determination systems for oviparous species only (n = 182 species), and ambient temperature in relation to the duration of the breeding season for oviparous species only (n = 182 species). Figure S3. World maps showing the distributions and average temperature of species with GSD or TSD and known breeding seasons. Figure S4. Ambient temperature and the variance within the geographic range of the species relative to the size of the geographic range of the species. Figure S5. Seasonality data from reptiles (n = 213) compared to random data. Figure S6. Correlations between continuous life history traits. [file 12862_2020_1671_MOESM1_ESM.pdf]

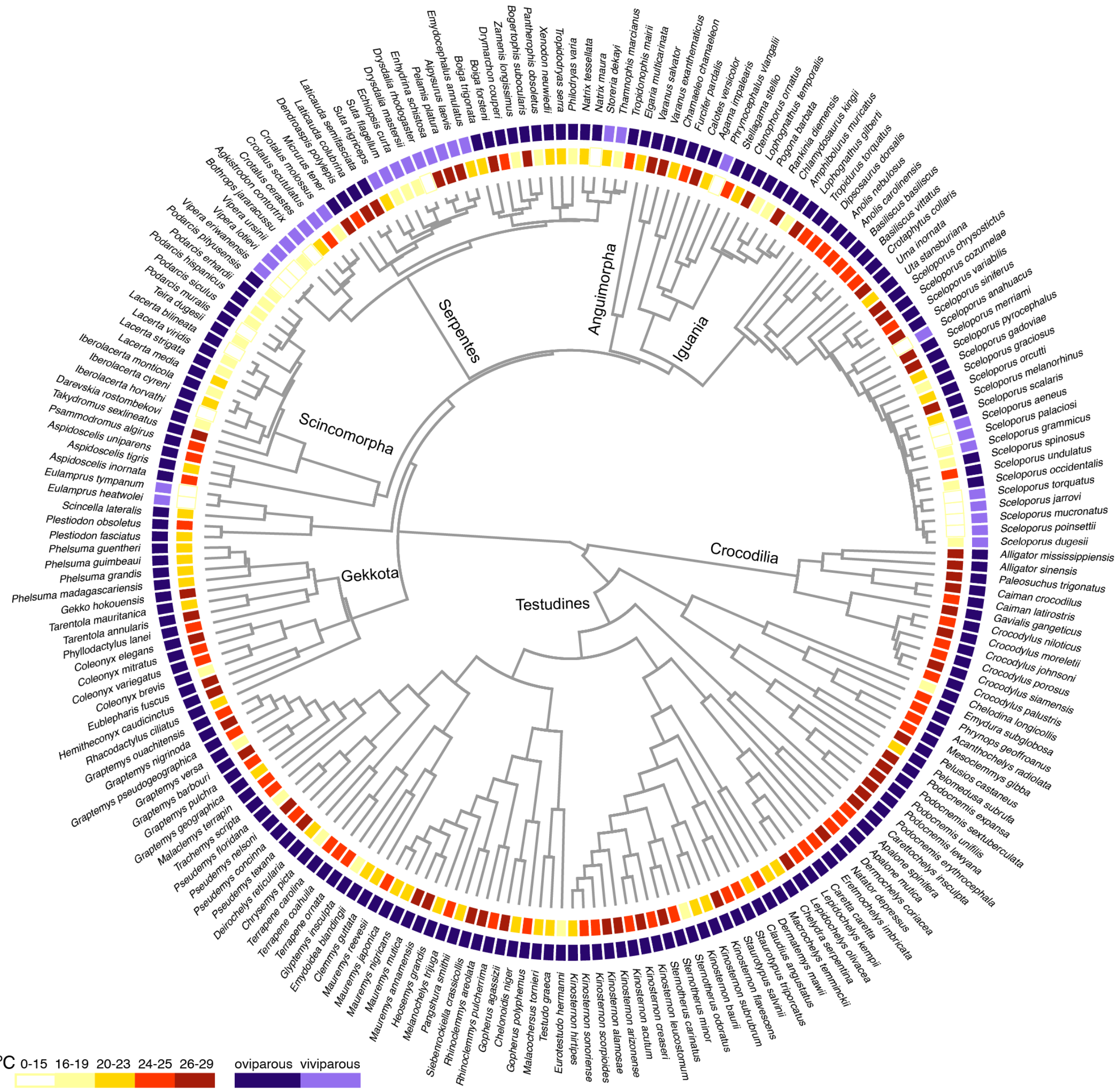

Figure S1. Phylogenetic relationship between reproductive mode and ambient temperature (N = 213 species). Relationships for 213 species with known breeding season (oviparous = 182 species, viviparous = 31 species). Dark purple and purple represent oviparous and viviparous species, respectively, whereas white, yellow, dark yellow, red, and dark red squares indicate annual ambient temperatures: very cold (0 – 15 °C), cold (16 – 19 °C), mild (20 – 23 °C), warm (24 – 25 °C), and very warm (26 – 29 °C), respectively. Names in the phylogenetic tree correspond to reptile orders or infraorders.

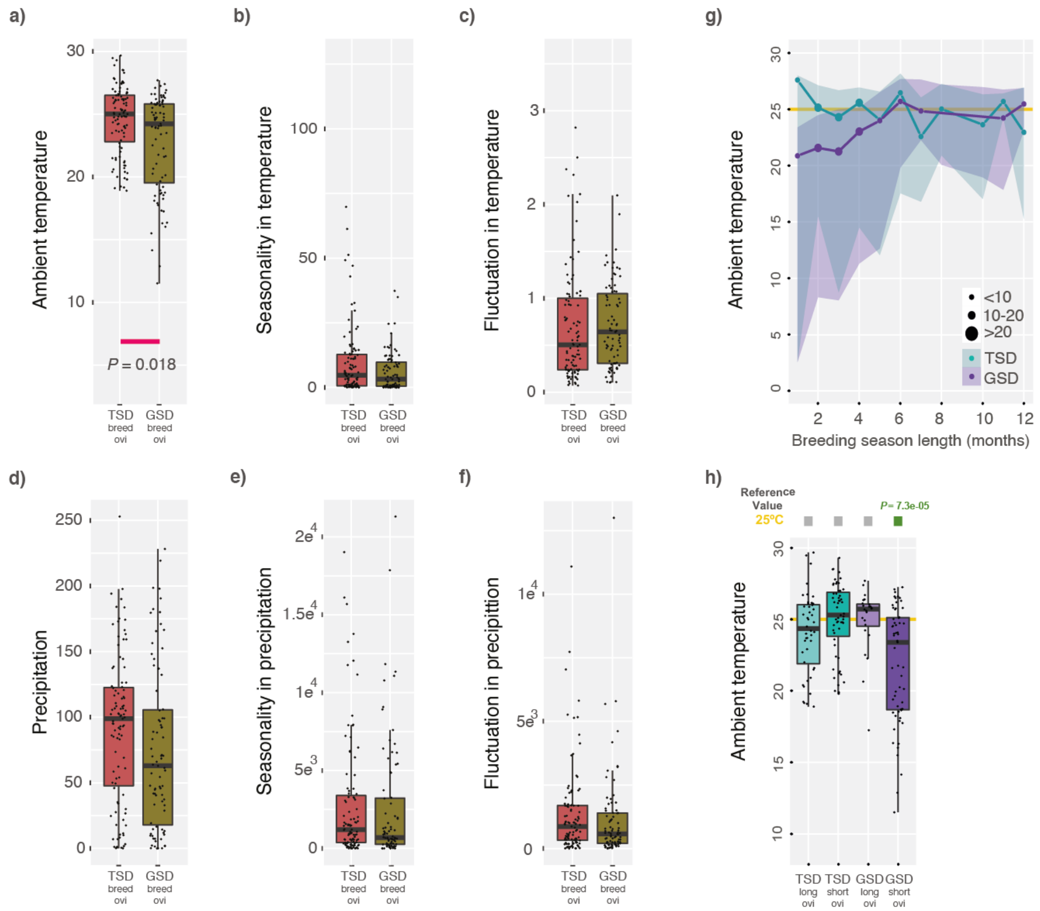

Figure S2. Climate and climatic fluctuations in reptiles with temperature-dependent and genotypic sex determination systems for oviparous species only (n = 182 species), and ambient temperature in relation to the duration of the breeding season for oviparous species only (n = 182 species). Boxplots representing **a)** ambient temperatures, **b)** seasonality in temperature, and **c)** between-year fluctuation in temperature for species with either TSD or GSD systems, based on breeding seasons estimates (breed) for oviparous species only (ovi). **d-f)** Similar to **a-c)** but for precipitation data. Significant differences (Phylogenetic Generalized Least Squares test): exact P values are indicated. Error bars, maximum and minimum values, excluding outliers. See Supplementary Tables 1-3 for details. Temperature is given in Celsius. Precipitation refers to ml units of rain. **g)** Dots indicate the average ambient temperatures for species with breeding seasons of different lengths (measured in months). GSD species are shown in purple, whereas TSD species are shown in green. The number of species included in each category is indicated by the size of the dots. Shaded areas represent the annual temperature range (average maximum and minimum temperatures) for the species in each category. The yellow line at 25 °C indicates the approximate average ambient temperature for the majority of groups. Temperature is given in Celsius. **h)** Boxplots representing ambient temperatures associated with TSD or GSD species with either long breeding seasons (5-12 months) or short breeding seasons (1-4 months). Significant differences (Mann-Whitney U test): Benjamin Hochberg-corrected *P* < 0.05 of temperatures against a reference value of 25 °C (shared average ambient temperature for the majority of groups in panel a); grey filled squares denote non-significant differences, whereas green filled squares denote significant differences (significant *P* values are indicated). Error bars, maximum and minimum values, excluding outliers. Temperature is given in Celsius.

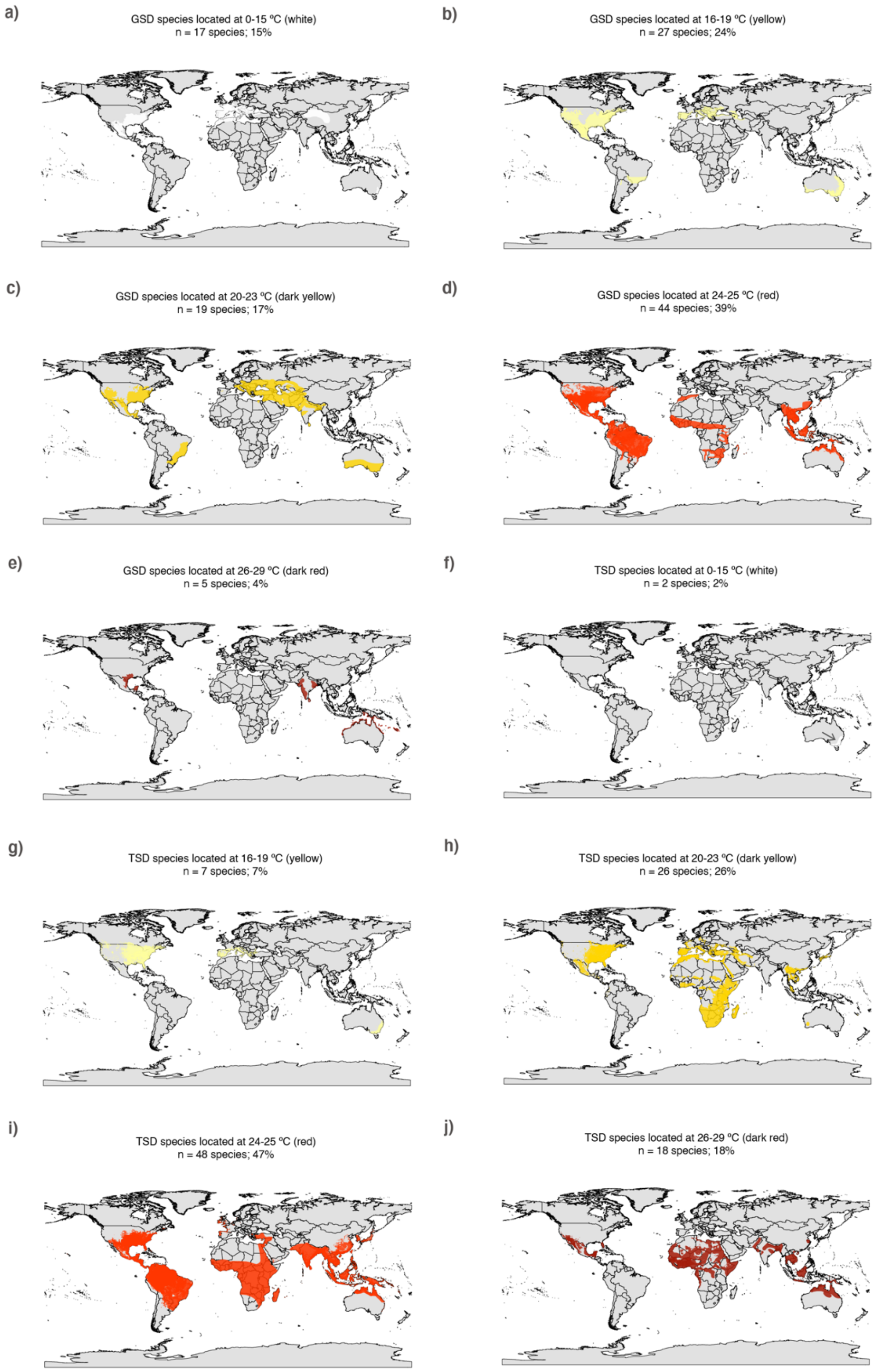

Figure S3. World maps of species with GSD or TSD and known breeding seasons. GSD species in **a)** very col (0 – 15 °C), **b)** cold (16 – 19 °C), **c)** mild (20 – 23 °C), **d)** warm (24 – 25 °C), and **e)** very warm (26 – 29 °C) ambient temperatures. TSD species in **f)** very cold (0 – 15 °C), **g)** cold (16 – 19 °C), **h)** mild (20 – 23 °C), **i)** warm (24 – 25 °C), and **j)** very warm (26 – 29 °C) ambient temperatures. The species' shapefiles were added onto the world map obtained from the R package *rworldmap* (Andy South, *The R Journal*. 2011. 3:1, pages 35-43).

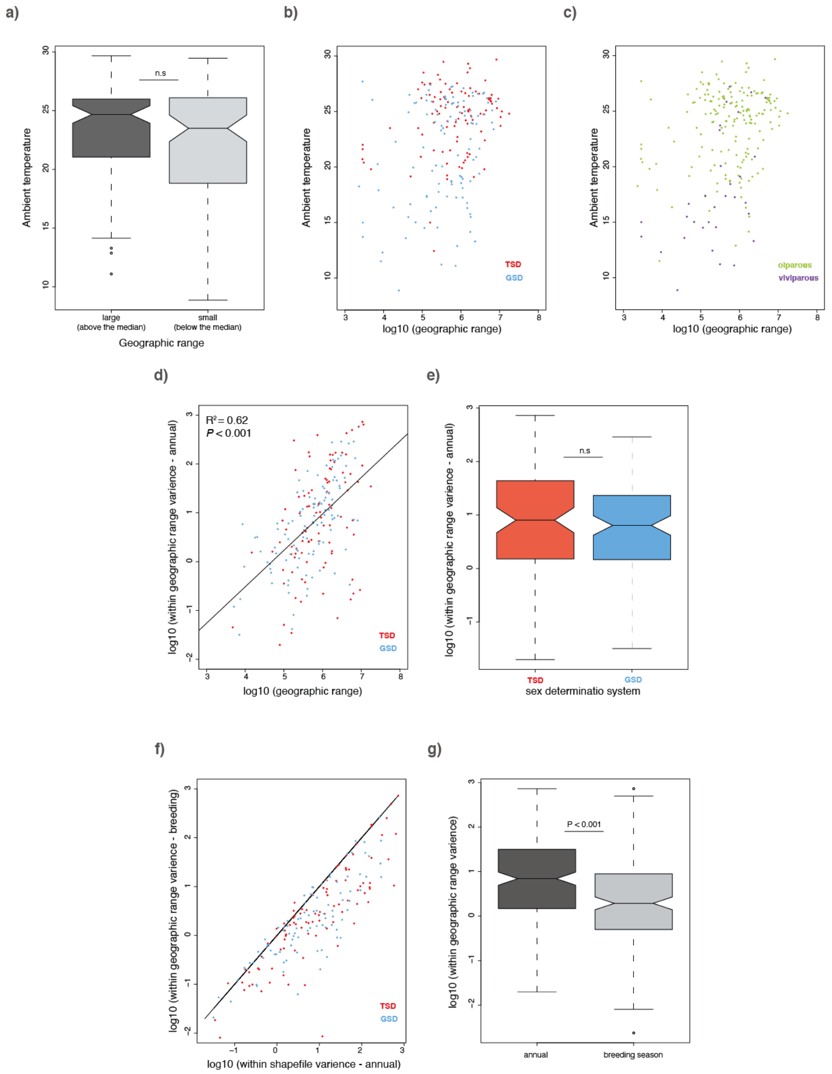

Figure S4. Ambient temperature and the variance within the geographic range of the species relative to the size of the geographic range of the species. a) Ambient temperature for species with large (above the median) and small (below the median) geographic ranges. **b)** Distribution of geographic ranges and their estimated averaged ambient temperatures for species with either TSD or GSD. **c)** Distribution of geographic ranges and their estimated averaged ambient temperatures for oviparous and viviparous species. **d)** Variance in temperature inside the geographic range of species with either TSD and GSD. **e)** Boxplots of the variance in temperature inside the geographic range of species with either TSD and GSD. **f)** Difference in the variance in temperature inside the geographic range of species with either TSD and GSD based on annual or breeding season estimates. **g)** Boxplots of the variance in temperature inside the geographic range of the species based on annual or breeding season estimates. Significant differences (Phylogenetic Generalized Least Squares test): exact *P* values are indicated. Error bars, maximum and minimum values, excluding outliers. Temperature is given in Celsius.

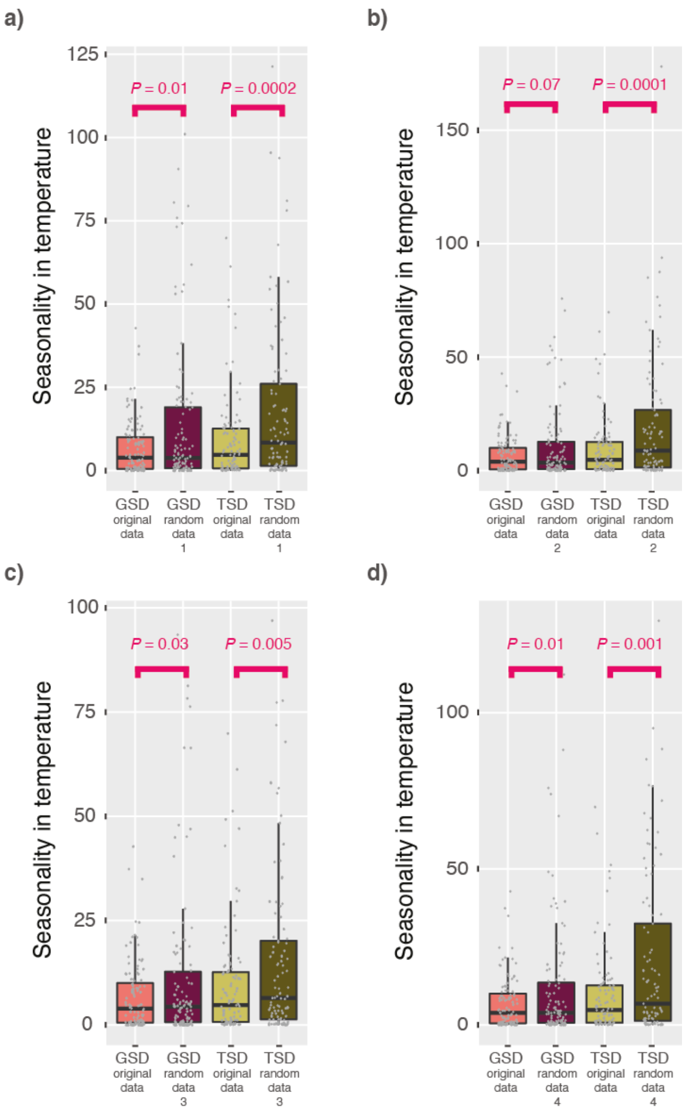

Figure S5. Seasonality data from reptiles (n = 213) compared to random data. a) Seasonality data for GSD and TSD species compared against a random set of temperature variations from contiguous months of the same length as the breeding seasons of each species. **b-d)** Same as a) for three additional random sets of temperature variations.

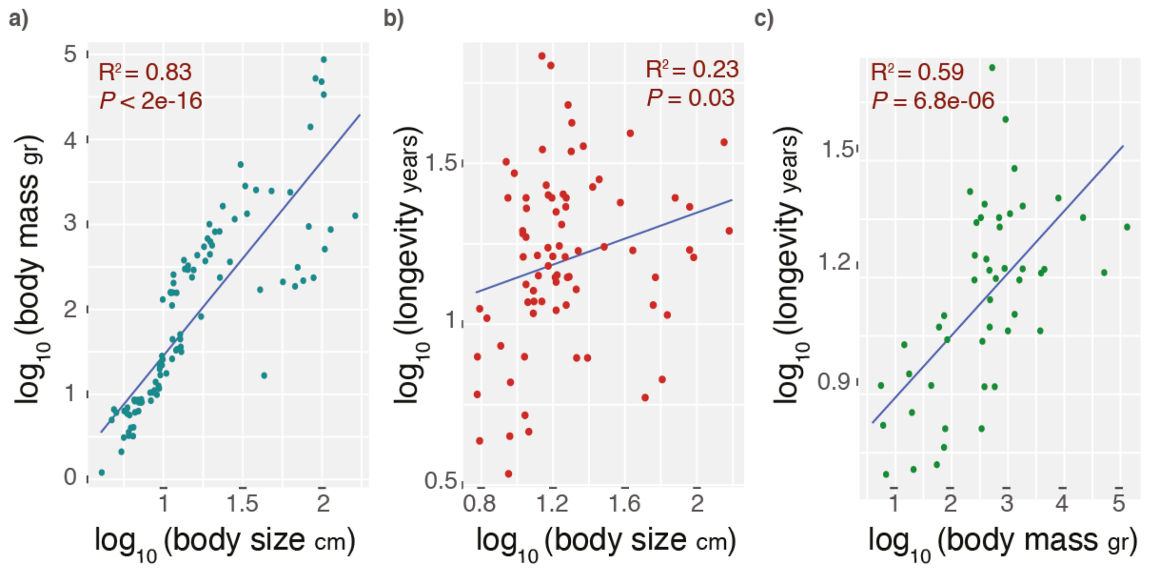

Figure S6. Correlations between continuous life history traits. a) Body size (average between sexes) against body mass (average between sexes). **b)** Body size (average between sexes) against longevity. **c)** Body mass (average between sexes) against longevity. Values were log10 transformed. R2 estimates and exact P values are indicated.
